# Adolescents Smoking in the Crosslight of Other Substance Use and Parental and Peers' Smoking Behaviors

**DOI:** 10.1155/2014/719681

**Published:** 2014-02-18

**Authors:** Hervé Kuendig, Marina Delgrande Jordan

**Affiliations:** Addiction Switzerland, P.O. Box 870, 1001 Lausanne, Switzerland

## Abstract

This study investigates the connectedness of adolescents' smoking status, history of alcohol and cannabis use, and parental and peers' smoking, dimensions only rarely explored concurrently. Multinomial regression models that compared the smoking status of adolescents were estimated based on a representative sample of 3,560 adolescents aged 14–15 from Switzerland. While 49.0% of respondents had never smoked, 9.0% smoked on a daily basis and 12.0% occasionally; 32.6% had never drank alcohol and 74.7% had never used cannabis. Overall, parental and peers' smoking and other substance use factors are significantly associated with smoking status. Yet, history of substance use revealed less consistent associations with smoking status among current smokers (daily versus occasional smoking). The findings highlight the connectedness of adolescents' and other substance use behaviors and support the relevance of concurrent prevention initiatives targeting adolescents with specific substance use profiles and/or growing up in prosmoking social milieus.

## 1. Introduction

Adolescents' smoking behavior is well documented in Switzerland [1–3]. The prevalence of smoking tobacco is comparable to the European average [4], although the age at consumption onset showed signs of decline over the last decade [3, 5]. While this observation might be in line with a slight decline of tobacco use among youth [2], it is significant as during adolescence nicotine dependence develops soon after smoking initiation and even from low levels of nicotine exposure [6–8].

Epidemiological studies have further highlighted that a consequent number of adolescents and young adults report using multiple psychoactive substances in Switzerland [9, 10]. For example, it was reported that smoking is strongly associated with alcohol consumption and to a lesser extent with cannabis use already among 15 years old [9]. Yet, the results from this latter Swiss study cover only few possible combinations of substance use patterns and do not consider factors related to adolescent smoking, such as social influences. Along with individual (e.g., other health-related behaviors, personality) and structural (e.g., purchase age restrictions, advertisement) factors, social factors, such as family and peer environment and particularly parental and peers' smoking, appear to have a considerable influence on adolescents' smoking behavior [11, 12]. Various theories, for example, social learning theory, primary socialization theory, social identity theory, and social network theory, provide a framework for understanding the social processes that play a role in adolescents' decisions to engage in smoking behavior [12–14]. While all of these theories emphasize a different social and cognitive process, they all converge in suggesting that norms and behaviors of adolescents' peers or family members are important determinants of (health-related) behavior. Hence, numerous epidemiological studies have documented the effects of other substance use behaviors on individual substance use, highlighting that peers' smoking is associated with smoking initiation, progression, and trajectory (see [14–16]). The effect of parental smoking on offspring's smoking was also repeatedly investigated in social risk-factors research, but associations have generally been modest and inconsistent across studies [17–20]. For instance, previous studies demonstrated that when considering these two influences concurrently, parents' substance use had an effect on adolescents' smoking although possibly weaker than peers' substance use [18, 21].

To our knowledge, no study explored adolescents smoking behaviors by investigating these dimensions concurrently. Thus, the lack of comprehensive vision of the interrelations among youth smoking, other substance use behaviors, and parental and peers' smoking can be seen as a flaw when trying to develop comprehensive (versus substance specific) preventive initiatives that can be (or not) universal or selective regarding targeted population and implementation setting [22–24].

The overall aim of the study is to document the association of selected factors (i.e., sociodemographic characteristics, other substance use experiences, and family and peers' smoking) with smoking behaviors among adolescents aged 14 and 15 years in Switzerland. More specifically, we aim at investigating the influence of these factors on adolescent smoking by contrasting their association with a hierarchy of smoking profiles.

## 2. Materials and Methods

We used data from the Swiss 2010 “Health Behaviour in School-Aged Children (HBSC)” survey [5], undertaken under the aegis of the World Health Organization (WHO-Europe). The study was granted ethical clearance by the Cantonal Ethics Committee for Research on Human Beings of Vaud canton (protocol 173/09) and conducted in accordance with APA ethical standards. The data were collected between January and April 2010 in classroom by means of a paper-and-pencil self-administered questionnaire (i.e., in French, German, or Italian). Classes (i.e. cluster sampling) were randomly selected proportionate to the size of the Swiss cantons. Pupils completed questionnaires independently over a 45 min school period (absent pupils at the time of data collection were not surveyed later on). Participation was voluntary and confidentiality guaranteed at all stages of the study. Classes participation rate reached 88.0%, while within participating classes pupils participation rate was of 94.8%. Detailed information about the study design can be found in Currie et al. [4] and Windlin et al. [5].

The following analysis was based on an original sample of 3,560 boys and girls of 14 and 15 years of age attending 8th and 9th grades drawn from a cluster sampling procedure of all 8th and 9th graders in Swiss public schools.

### 2.1. Measures

An interdisciplinary research group developed the questionnaire, including optional items on family and peer substance use [1, 25].

### 2.2. Dependent Variable: Adolescent Smoking Status

Smoking status was defined based on two questions investigating smoking experience and behaviors; that is, “have you ever smoked tobacco?” (response options “yes/no”) and “how often do you smoke tobacco at present?” (options: every day/at least once a week but not every day/less than once a week/I do not smoke). Based on these items, smoking status was categorized, resulting in the following four smoking experience profiles: *lifetime abstinence* (never smoker), *current abstinence* (ever smoker but not at present), *current occasional smoking* (weekly or more seldom but not daily), and *current daily smoking*.

### 2.3. Independent Variables: Alcohol and Cannabis Use, Family and Peers' Smoking, and Sociodemographics

*Alcohol Use (3 Categories).* Alcohol use indicator was created based on inquiring about whether the respondent drank alcoholic beverages and, if yes, the number of drinks he/she was drinking on a typical drinking occasion. Based on their responses, youths were categorized as “*never drinker*” (i.e., individuals who never had drunk alcohol), “*light drinker*” (i.e., drinking less than one drink per occasion), and “*drinker*” (i.e., drinking one or more drink per occasion).

*Cannabis Use (3 Categories). *Three items assessing the frequency of use of cannabis over the last 30 days, the last 12 months, and ever, respectively (response options ranging from “never” to “40 times or more”), were used to examine experimenting with or smoking cannabis. Respondents were categorized as *never user* (no report of any episode of use), *experimenter* (i.e., who smoked “1-2 times,” independently of the timeframe), and *user* (i.e., report of more than two episodes of use).

*Parental Smoking (3 Categories)*. Whether youths' parents were smokers was assessed by the question, “does anyone in your family smoke?,” for which the pupils were asked to indicate if their “dad (step-dad or mom's partner)” and “mom (step-mom or dad's partner)” were smoking. Multiple response options were coded into three categories, which are none of the parents (or analogous parental figures) smoked, one of them smoked, or both smoked.

*Peers' Smoking*. The exposure to peers' smoking was based on respondents' response to a question, “According to your opinion, how many of your friends smoke?”. Response categories were here considered on a continuum ranging from “none of them” to “all of them” (intermediate option being “few of them,” “about half of them,” and “the majority”; Peers' smoking was considered either as continuous, scale ranging from “0” to “4,” or asll categorical; see Section 2.5 for more details).

*Sociodemographics.* Gender (female as reference) and age (14 versus 15 years; continuous) were further considered as these variables are important predictors of trying out or developing substance use behaviors in adolescence.

### 2.4. Final Sample Description

Regarding the questions on adolescent smoking, 0.6% of values were missing. Missing values for indicators related to history of alcohol and cannabis use as well as family and peers' smoking ranged from 0.8% to 1.3%. Youths with any missing data on these indicators (4.6%) were excluded from the analysis. The final sample consists of 3,395 pupils (1,639 boys and 1,756 girls) aged 14 and 15 (mean: 15.00; SD: .54; decimal year).

### 2.5. Analytical Strategy

Relationships among smoking status and history of alcohol and cannabis use, parental and peers' smoking, and sociodemographics were estimated using multinomial regression models with different smoking status condition as a reference group. The presented analyses considered smoking status as dependent variable and *alcohol use* (treated as categorical, *never drinker* as reference), *cannabis use* (*never user* as reference), *parental smoking *(*no parents smoking* as reference), and *peers' smoking* (treated both as continuous and categorical; see the scale description above), as well as *gender* (*male* as reference) and *age *(continuous) as independent factors. Interaction effects between parental and peers' smoking on adolescent smoking were tested in an additional set of multinomial regression models (results not presented in table). Peers' smoking was then treated as categorical, as were the interaction terms.

All analyses were performed using the statistical software package STATA SE 11.2 [26] and were adjusted for cluster sampling design effects.

## 3. Results

Distributions of smoking status, alcohol and cannabis use, family and peers' smoking indicators by *gender* and *age* are presented in Table 1. In total, 49.0% of youth aged 14 to 15 reported to have never smoked in their lifetime, 9.0% reported smoking on a daily basis, and 12.0% reported smoking less frequently. The remaining 30.0% were current abstainers (i.e., ever smoker but not at present). *Current daily smoking* appeared to increase markedly between the age of 14 (6.1%) and the age of 15 (11.6%); *current occasional smoking* remained rather constant. In terms of alcohol use, more than 60% of 14-year-old subjects were *never drinkers* or *light drinkers,* but almost 55% of 15-year-old subjects were *drinkers*. Moreover, more than one out of four respondents reported using cannabis use. Specifically, 10.0% had used cannabis once or twice (*cannabis experimenters*) and 15.2% used it more often (*cannabis users*). Finally, with regard to parental and peers' smoking, 40.8% of respondents reported that at least one of their parents smoked, and 37.9% indicated that about half or more of their friends smoked (i.e., about half, majority, or all friends smoked).

Distribution of substance use (alcohol and cannabis) and of parental and peers' smoking given the smoking status of the respondents can be found in Table 2. While a majority of youth who had never been smoking had never been drinking either (54.2%), respectively, 59.9% of current abstainers, 82.9% of *occasional smokers*, and 90.0% of *current daily smokers* were classified as drinker. Also, while 97.7% of *lifetime abstainers* (smoking) had never tried cannabis, 84.4% of *daily smokers* had (11.5% of cannabis experimenters and 72.9% of users).

The results from the set of multinomial regression models can be found in Table 3. Comparing *lifetime *and *current abstinence*, other substance use behaviors as well as family and peers' smoking were significantly associated with smoking status. Being a *light drinker* and/or *cannabis experimenter* and being a *drinker* and/or *cannabis user* were positively associated with having tried smoking. Parental and peers' smoking were also positively associated with it. Specifically, more intensive use of alcohol and/or cannabis was associated with a greater likelihood of being *current *versus* lifetime *abstainer (i.e., coefficient increased with the rise in the use or exposure). Besides, the higher parental and/or peers' smoking exposure, the greater likelihood to having tried smoking. For crude interpretation, based on the reported coefficients, *drinkers* had more than five times higher probability of trying smoking compared to someone who has never drank alcohol (i.e., *e* ^1.64^ = 5.16).

Similar observations came from comparing other smoking status conditions with each other. Consistently, all substance use and smoking exposure conditions loaded positively with the more intensive smoking status. Further, the magnitude of loadings concurred with the intensity of exposition to independent factors. In every model, *drinkers* and *cannabis users* had larger loadings compared to *light drinkers* and *cannabis experimenters*, and youth with both parents smoking had larger loadings than did youth with one parent smoking. Furthermore, coefficients relating to peers' smoking loaded positively on smoking status, indicating that the likelihood of more intensive smoking behaviors increased with the proportion of smoking peers.

With the exception of two coefficients reaching only borderline levels of significance (i.e., *P value*s of 0.069 for the associations between having *one* and *two parents smoking* and *current occasional smoking* versus *current abstinence*), all measures of association between parental and peers' smoking factors and smoking status reached significance. This contrasts with a kind of backward influence effect of other substance use measures and particularly of alcohol use on smoking status. This influence is specifically observed in the fact that, whereas all considered condition associated significantly with smoking status when contrasting *lifetime abstinence* with the other status, being *light drinker* (versus having never tasted alcohol) was not associated with smoking status in other comparisons. In the same way, being a cannabis experimenter (versus having never smoked cannabis) was not associated with smoking status when contrasting *current occasional *versus* daily smoking* but was in another instance.

Finally, considering the effects of other substance use and smoking environment factors, *gender* and *age* were inconsistently associated with smoking status. Whereas negative coefficient loadings were consistently observed for *gender* (i.e., females being apparently less inclined to smoke or smoke regularly compared to males) the association did not reach significant level in any of the six status comparisons. Regarding *age*, the results are less consistent but reveal differences that are of relevance when describing factors involved in the progression of smoking behaviors. *Current daily smoking* showed positive loading of *age* compared to other conditions (and even significant positive association when compared to *current occasional smoking*, that is, indicating that older age is associated with more intensive smoking among smokers). Yet, when comparing the two groups of abstainers with *current occasional smoking, age* associated negatively with *current occasional smoking*, showing that, when controlling for the effect of other substance use behaviors and environmental factors, getting older was linked to nonsmoking behaviors among youths who do not smoke on a daily basis).

Additional multinomial regression models were performed to investigate potential interaction effects between parental and peers' smoking on individual smoking status. The results revealed nonsignificant interaction. Further, the direction and significance level of associations documented in Table 3 did not vary meaningfully in these models (detailed results can be obtained from the authors upon request).

## 4. Discussion

The aim of this study was to investigate the association of adolescents' smoking behaviors with alcohol and cannabis use and parental and peers' smoking while taking into account gender and age effects. Based on a large representative sample of 14 and 15 years of age, our results highlight high smoking rates among youth of this age in Switzerland and suggest that parental and peers' smoking behaviors, as well as adolescents own alcohol and cannabis use, are highly associated with adolescents' smoking status.

Compared to adolescents who had never drank alcohol in their life (or at least not more than a sip or so), light drinkers (drinking less than 1 drink per occasion) were more likely to have ever tried smoking and to currently smoke (versus never have smoked). This likelihood was even higher for youth drinking usually one or more drinks per drinking occasion. Similar associations were observed between cannabis use and smoking. These results are in line with previous studies that have reported clustering of risk behaviors during adolescence [27]. Yet, noteworthy is the observation that being a light drinker (versus never drinker) was associated with having or not tried to smoke but not with more extended smoking experience, while drinking more intensively was further associated with current smoking behavior (i.e., positive association with occasional and daily smoking versus current abstinence). This accordingly suggests a possible dose response effect in the association between substance use behaviors and experience, more specifically among smoking status, alcohol, and cannabis use. This provides additional evidence of the relevance of developing multisubstance and/or multirisk behaviors prevention programs [28].

Regarding parental smoking, about two out of five adolescents reported that at least one of their parents smoked. These youths had higher likelyhoods to smoke and to smoke on a daily basis compared to those whose parent did not smoke. This likelihood was higher for youths with both parents smoking than for those with only one smoking parent. This suggests that the exposure to parental smoking increases the likelihood that adolescents will start using tobacco and will smoke intensively if they are going to smoke. This finding is consistent with the literature on parental influence on offspring smoking behaviors (see, e.g., [29]). Several hypotheses can explain the underlying mechanisms of parental influence. Environmentally, for example, parental smoking may directly influence smoking in offspring through behavioral modeling and permissiveness toward smoking and/or indirectly through positive attitudes toward smoking and perceived availability of tobacco [29, 30]. Further exploration of this hypothesis would help determine which of these domains should be targeted as a priority in future preventive strategies aimed at reducing concurrently parental and offspring smoking, for example.

Our study conducted with 14-and-15 year-old adolescents in Switzerland also confirms one of the most robust findings in the literature on adolescent smoking risk factors, that is, the association between adolescents' and peers' smoking (see, e.g., [12, 14]). Specifically, the findings indicate that adolescents who have more smoking peers are more likely to be smoking and smoking daily. The HBSC's cross-sectional study showed the tendency for adolescent peer group members to share common characteristics, like smoking, but did not allow concluding that peers' smoking (directly) influences adolescent smoking. Actually, both socialization effect, which refers to the tendency to be influenced by effective or perceived attitudes and behaviors of one's friends, and selection effect, which refers to the tendency to join people with similar attitudes and behaviors, may contribute to peer group homogeneity [14].

Finally, despite the adolescent's growing autonomy from parents and increased orientation towards peers, our findings indicate that, while peers appear to have a strong influence on adolescents' smoking behavior, parents' influence remains significant at the age of 14-15, at least from a statistical point of view. However, the cross-sectional nature of the study does not give opportunities to assess how and when parental and peers' smoking contribute to this behavior; for example, whether pathways exist among parental smoking, affiliation with peer networks where smoking is widespread, and adolescents own smoking behaviors [31]. Furthermore, the study could not determine whether the homogeneity of smoking behaviors within a given peer group should be considered through peer socialization process, which might only be indirectly influenced by the larger social context, including parental smoking [12].

A number of additional limitations should be noted. One limitation relates to the fact that the measure of peer smoking is nonspecific. It does not differentiate between more (i.e., best friends) and less significant friends and does not differentiate same-sex and opposite-sex peers' smoking, whereas the closeness and status of smoking peer might exert different influences on adolescent smoking [12]. Additionally, the item assessing peer smoking is based on a subjective report of friends' behavior and, to some extent, it can be seen as a proxy assessment of the perception of smoking norm, the latter being another factor influencing smoking behaviors during adolescence [22]. Further, parental smoking measurements distinguish only between smokers and nonsmokers. Yet, current nonsmoking status does not consider the influence of past behaviors; that is, whether a non-smoking parental figure was actually a former smoker (who possibly quit smoking only recently) or never smoker. This lack of specificity might imply bias. Yet, since the effect size of estimates of association between parental and youth smoking should be weakened, the reported estimates can be considered conservative. Also, the fact that data collection is based on self-reported measures collected within school classes might imply both desirability bias (i.e., either over- or understatements of risk behaviors) and clustering effect. Whereas it is impossible to control or assess the former, the effects of the cluster sampling design were taken into account within the analytical strategy applied. Other limitations relate to the measurement tools applied in this study. First, since the measure of smoking behavior did not address the quantity of cigarettes smoked, for example, among current daily smokers or among current abstainers, a detailed history of past and current tobacco use pattern could not be established. Future studies on early adolescence “life course” changes of smoking status considering other substance use and parental and peer influence should be aimed at. Also, more detailed measures of drinking patterns (e.g., considering not only usual quantities of alcohol consumed, but also the frequency of consumption and the propensity to heavy episodic drinking) as well as additional sociodemographic covariates could be considered.

In conclusion, our findings can be considered in the development of future substance use prevention initiatives. They highlight the strong intricacy of the association of adolescent smoking behaviors with other substance use behaviors and parental and peers' smoking behaviors. Accordingly, they support the relevance of concurrent prevention initiatives in Switzerland that could target adolescents with specific substance use profile and/or those who are growing up in social milieus—families and peer networks—where smoking is possibly not seen as an issue.

## Figures and Tables

**Table 1:** Distribution of substance use status (smoking status, alcohol use, and cannabis use) and parental and Peers' smoking by gender, age, and total.

	Gender				Age					
	Males		Females		14		15		Total	
	*n*	%	*n*	%	*n*	%	*n*	%	*n*	%
Smoking status										
Lifetime abst.	756	46.1	909	51.8	893	54.3	772	44.1	1665	49.0
Current abst.	508	31.0	511	29.1	465	28.3	554	31.6	1019	30.0
Occas. smo.	211	12.9	196	11.2	185	11.3	222	12.7	407	12.0
Daily smo.	164	10.0	140	8.0	101	6.1	203	11.6	304	9.0
Alcohol use										
Never drinker	537	32.8	571	32.5	638	38.8	470	26.8	1108	32.6
Light drinker	309	18.9	392	22.3	373	22.7	328	18.7	701	20.6
Drinker	793	48.4	793	45.2	633	38.5	953	54.4	1586	46.7
Cannabis use										
Never user	1150	70.2	1387	79.0	1316	80.0	1221	69.7	2537	74.7
Experimenter	177	10.8	164	9.3	145	8.8	196	11.2	341	10.0
User	312	19.0	205	11.7	183	11.1	334	19.1	517	15.2
Parental smoking										
None	984	60.0	1025	58.4	1018	61.9	991	56.6	2009	59.2
One	464	28.3	493	28.1	433	26.3	524	29.9	957	28.2
Both	191	11.7	238	13.6	193	11.7	236	13.5	429	12.6
Peers' smoking										
None (0)	457	27.9	530	30.2	563	34.2	424	24.2	987	29.1
Few (1)	551	33.6	570	32.5	571	34.7	550	31.4	1121	33.0
About half (2)	338	20.6	345	19.6	273	16.6	410	23.4	683	20.1
Majority (3)	225	13.7	271	15.4	197	12.0	299	17.1	496	14.6
All (4)	68	4.1	40	2.3	40	2.4	68	3.9	108	3.2

Total *N*	1639		1756		1644		1751		3395	

**Table 2:** Distribution of substance use status (alcohol use and cannabis use) and parental and peers' smoking by smoking status.

	Smoking status							
	Lifetime abst.		Current abst.		Occas. smo.		Daily smo.	
	*n*	%	*n*	%	*n*	%	*n*	%
Alcohol use								
Never drinker	926	54.2%	185	17.7%	21	4.9%	7	2.2%
Light drinker	409	23.9%	235	22.4%	52	12.2%	25	7.8%
Drinker	373	21.8%	628	59.9%	353	82.9%	287	90.0%
Cannabis use								
Never user	1683	97.7%	715	68.0%	175	41.1%	49	15.6%
Experimenter	29	1.7%	191	18.2%	98	23.0%	36	11.5%
User	10	0.6%	146	13.9%	153	35.9%	229	72.9%
Parental smoking								
None	1176	68.7%	592	56.4%	206	48.8%	97	30.6%
One	391	22.9%	323	30.8%	145	34.4%	127	40.1%
Both	144	8.4%	134	12.8%	71	16.8%	93	29.3%
Peers' smoking								
None (0)	798	47.0%	193	18.5%	8	1.9%	7	2.2%
Few (1)	589	34.7%	432	41.5%	100	23.6%	21	6.7%
About half (2)	214	12.6%	280	26.9%	148	34.9%	55	17.5%
Majority (3)	82	4.8%	123	11.8%	146	34.4%	168	53.5%
All (4)	16	0.9%	14	1.3%	22	5.2%	63	20.1%

**Table 3:** The results of the set of multinomial logistic regression models contrasting different smoking status. Smoking status regressed on other substance use, parental and peers' smoking indicators, gender, and age. Models accounting for cluster design effects: regression coefficients (*b*), standard error (SE), *t*-values and significance level (sig.).

Contrast condition	*Lifetime abstinence *			*Current abstinence *			*Occasional smoking *		
	*b*	SE	*t* sig.	*b*	SE	*t* sig.	*b*	SE	*t* sig.
*Daily smoking *									
Alcohol^†^									
Light d.	1.59	0.52	3.07∗∗	0.66	0.51	1.29^ns.^	0.21	0.55	0.38^ns.^
Drinker	3.07	0.43	7.17∗∗∗	1.43	0.43	3.33∗∗	0.31	0.47	0.66^ns.^
Cannabis^†^									
Exp.	3.11	0.34	9.19∗∗∗	0.85	0.28	3.05∗∗	0.30	0.29	1.02^ns.^
User	5.16	0.41	12.65∗∗∗	2.39	0.21	11.22∗∗∗	1.48	0.22	6.71∗∗∗
Parental smoking^†^									
One	1.53	0.20	7.61∗∗∗	1.06	0.18	5.79∗∗∗	0.80	0.18	4.37∗∗∗
Two	1.88	0.26	7.30∗∗∗	1.37	0.23	5.94∗∗∗	1.05	0.22	4.68∗∗∗
Peer smo.^‡^	1.71	0.13	13.33∗∗∗	1.34	0.12	11.17∗∗∗	0.64	0.11	5.72∗∗∗
Gender	−0.20	0.19	−1.03^ns.^	−0.11	0.17	−0.61^ns.^	−0.05	0.17	−0.26^ns.^
Age	0.02	0.19	0.09^ns.^	0.08	0.18	0.42^ns.^	0.36	0.18	2.00∗
*Occasional smoking *									
Alcohol^†^									
Light d.	1.38	0.30	4.66∗∗∗	0.45	0.31	1.48^ns.^			
Drinker	2.76	0.26	10.55∗∗∗	1.12	0.27	4.20∗∗∗			
Cannabis^†^									
Exp.	2.81	0.24	11.54∗∗∗	0.55	0.16	3.46∗∗			
User	3.69	0.37	9.86∗∗∗	0.92	0.18	5.15∗∗∗			
Parental smoking^†^									
One	0.73	0.16	4.58∗∗∗	0.27	0.15	1.83^ns.^			
Two	0.83	0.21	3.97∗∗∗	0.32	0.17	1.82^ns.^			
Peer smo.^‡^	1.08	0.07	15.28∗∗∗	0.70	0.06	11.26∗∗∗			
Gender	−0.15	0.14	−1.06^ns.^	−0.06	0.13	−0.45^ns.^			
Age	−0.34	0.16	−2.17∗	−0.29	0.14	−2.08∗			
*Current abstinence *									
Alcohol^†^									
Light d.	0.92	0.13	7.36∗∗∗						
Drinker	1.64	0.12	14.12∗∗∗						
Cannabis^†^									
Exp.	2.26	0.21	10.88∗∗∗						
User	2.77	0.33	8.32∗∗∗						
Parental smoking^†^									
One	0.47	0.11	4.33∗∗∗						
Two	0.51	0.16	3.26∗∗						
Peer smo.^‡^	0.37	0.05	7.14∗∗∗						
Gender	−0.09	0.10	−0.92^ns.^						
Age	−0.06	0.10	−0.57^ns.^						

Remarks: ^†^reference categories: alcohol: never drinker (versus light drinker and drinker), cannabis: never user (versus experimenter and user), and parental smoking: no parent smoke (versus one and two); ^‡^peer smoking is considered continuous (scale 0 to 4); ∗*P* < 0.05;∗∗*P* < 0.01; ∗∗∗*P* < 0.001; ns.: not significant at *α* = 5% level.
